# Practical tips and new trends in electrochemical biosensing of cancer-related extracellular vesicles

**DOI:** 10.1007/s00216-023-04530-z

**Published:** 2023-01-22

**Authors:** Patrick Severin Sfragano, Serena Pillozzi, Gerolama Condorelli, Ilaria Palchetti

**Affiliations:** 1 Department of Chemistry Ugo Schiff, University of Florence, Via Della Lastruccia 3, 50019 Sesto, Fiorentino, Italy; 2 Medical Oncology Unit, Careggi University Hospital, Largo Brambilla 3, 50134 Florence, Italy; 3 Department of Molecular Medicine and Medical Biotechnology, Federico II University of Naples, Via Pansini, 5, 80131 Naples, Italy; 4 IRCCS Istituto Neurologico Mediterraneo (INM) Neuromed, Via Atinense 18, 86077 Pozzilli, Italy

**Keywords:** Extracellular vesicle, Exosome, Electrochemical biosensor, Photoelectrochemical biosensor, Microfluidics, miRNA

## Abstract

To tackle cancer and provide prompt diagnoses and prognoses, the constantly evolving biosensing field is continuously on the lookout for novel markers that can be non-invasively analysed. Extracellular vesicles (EVs) may represent a promising biomarker that also works as a source of biomarkers. The augmented cellular activity of cancerous cells leads to the production of higher numbers of EVs, which can give direct information on the disease due to the presence of general and cancer-specific surface-tethered molecules. Moreover, the intravesicular space is enriched with other molecules that can considerably help in the early detection of neoplasia. Even though EV-targeted research has indubitably received broad attention lately, there still is a wide lack of practical and effective quantitative procedures due to difficulties in pre-analytical and analytical phases. This review aims at providing an exhaustive outline of the recent progress in EV detection using electrochemical and photoelectrochemical biosensors, with a focus on handling approaches and trends in the selection of bioreceptors and molecular targets related to EVs that might guide researchers that are approaching such an unstandardised field.

## Introduction

**Fig. 1:**
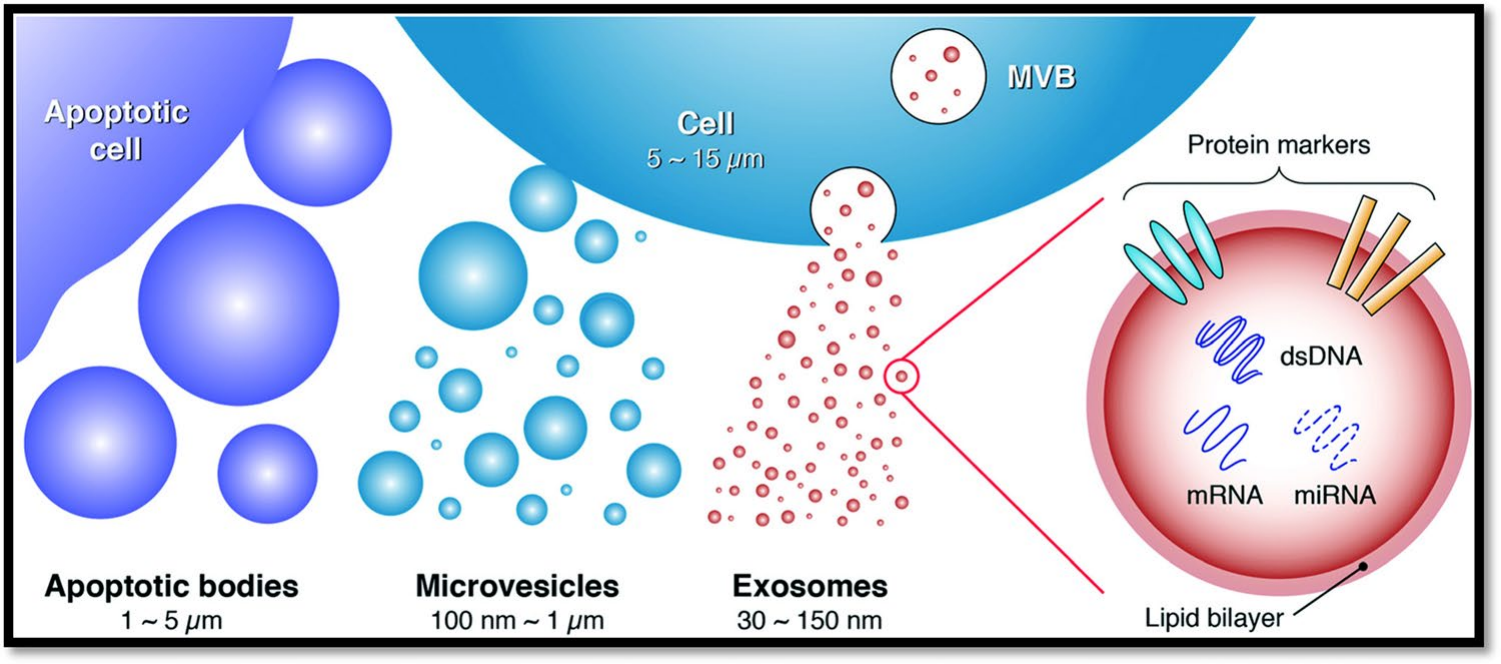
One possible classification of EVs on the basis of their size and biogenesis: apoptotic bodies originate from apoptotic cells, microvesicles bud off the plasma membrane of cells, and exosomes emerge from endosome-multivesicular body (MVB) complexes. Moreover, EVs are characterised by protein markers on their surface, as well as intravesicular nucleic acids, including double-stranded DNA (dsDNA), messenger RNAs (mRNAs), and microRNAs (miRNAs). Reprinted with permission from ref. [9]

Liquid biopsy has provided, over the years, pivotal support in the clinical field due to the remarkable advantages that it covers, including the possibility of obtaining cell-specific biomarkers from biological fluids with simple and easily repeatable techniques [1]. Among the variety of molecules that flow in biological fluids, circulating tumour cells (CTCs), microRNAs (miRNAs), long non-coding RNAs (lncRNAs), and vesicles are just a few of the most interesting and commonly studied. Regarding the latter, initially under-appreciated and considered simple debris or ways to dispose of cellular components [2], extracellular vesicles (EVs) are membrane-enclosed cell-secreted nanometric particles now considered biomarkers and, at the same time, sources of biomarkers [3]. Many types of these nanometric particles can be found, but their classification appears not to be unique. One possible distinction is among (*i*) microvesicles (particles with dimensions around 100–1000 nm, produced due to imbalances in the lipid distribution on plasma membranes), (*ii*) ectosomes (ubiquitous vesicles generated at the plasma membrane), (*iii*) shedding vesicles, and (*iv*) apoptotic bodies (100–5000 nm, secreted by apoptotic cells) (Fig. 1) [4]. Nevertheless, other classifications may be found in the literature, for instance on the basis of their size: (*a*) large exosomes: 90–120 nm, (*b*) small exosomes: 60–80 nm, and (*c*) exomeres (non-membranous EVs): 35 nm [5]. Other sources categorise them by their excretion mechanism; in particular, (*I*) large EVs or microvesicles are particles that bud from cells’ plasma membrane, whereas (*II*) small EVs or exosomes are multivesicular endosomes that release intraluminal vesicles upon fusing with the plasma membrane [6]. However, all these categories have evolved over the years and are still confusing.

Most research papers focus on exosomes. Nonetheless, there are some nomenclature issues regarding these vesicles, and the term “exosome” is often found in the literature to indicate any EV in general [7]. To this end, the MISEV2018 guidelines suggest researchers to use instead the more general term “extracellular vesicles” to avoid misunderstandings in scientific publications or use other parameters or terms to refer to the vesicles analysed, such as descriptors relative to their biochemical composition or their physical characteristics, including size (small, medium, large) or density (low, middle, high) [8]. Hence, to avoid possible confusion and misinterpretations, the generic term “extracellular vesicle” will be used in this manuscript, even when the scientific papers herein reported state otherwise.

In the last decade, multiple techniques have been used to detect and/or characterise EVs, including western blotting, mass spectrometry, flow cytometry, nanoparticles tracking analysis, dynamic light scattering, tunable resistive pulse sensing, transmission electron microscopy, atomic force microscopy, and enzyme-linked immunosorbent assays [10–13]. However, many of these approaches entail limitations, such as the high cost of the sophisticated instruments required, the incompatibility with in situ analyses, the lack of standardisation in the case of EVs, and the fact that large volumes of samples are often required [14]. An easier and cost-effective solution is lately increasingly being found in the field of biosensors. With their numerous virtues and their versatility toward point-of-care applications [15], many biosensing approaches have been challenged in EV analysis, spanning from (*a*) optical biosensors, based on colorimetry, fluorescence, chemiluminescence, surface plasmon resonance, surface-enhanced Raman spectroscopy, or photonic structures, to (*b*) electrochemical and photoelectrochemical biosensors that convert the biorecognition event between a receptor and its ligand into potential-, current-, or impedance-based signals by voltametric/amperometric readouts or electrochemical impedance spectroscopy (EIS). Indeed, (photo)electrochemical approaches are constantly evolving toward portability and miniaturisation [6], and lab-on-a-chip structures and wearable tools, that are compact and portable, might represent the greatest next generation of biosensing devices in the clinical field. Numerous review papers have been published in the last few years covering a summary of the recent strides taken in the field of biosensors using a variety of techniques [6, 16–29]. Herein, instead, the reader will find a focus on electrochemical and photoelectrochemical biosensing strategies, with the aim of giving a guide to the recent procedures found in the literature, as well as precautions and handling of EVs that may be of help to anyone that wants to approach the field, which is indeed constantly evolving and not yet standardised.

## Handling extracellular vesicles

### Storage conditions

The stability of extracellular vesicles is one of the main hurdles that hinder their analytical application. Moreover, the temperature at which they are stored has important outcomes. However, although new insights on EV preservation are emerging, currently there is a lack of standard criteria for better storage [30]. Storage is pivotal both for the EV-containing matrix, from which EVs are harvested, and for isolated EVs, affecting their stability and the number of vesicles retrievable [31]. Drastic size changes have been observed in EVs subjected to freeze–thaw cycles [32]. Freezing processes can induce the formation and expansion of ice micro- and nano-crystals in the lipid bilayer, thus disrupting the membrane. The value of the zeta potential (*ζ*) can thus be affected, resulting in its decrease and the formation of multilamellar vesicles and aggregation upon lowering the temperatures [33]. Vesicles stored at a wide range of storage temperatures have been compared, observing alterations in the vesicle’s diameter [17, 33, 34] and major protein and RNA losses at > 0 °C [35]. However, such results do not always arrive at the same conclusions but agree on the fact that, since EVs and their content are relatively unstable, it is recommended to store them below −70 °C. Indeed, more studies need to be conducted in this field, especially regarding short-time effects of sub-zero temperatures [30]. An additional parameter that should be carefully considered is pH. Unfortunately, very little information can be found on this matter in the literature. It has been shown that, upon isolation, a slightly basic pH resulted in a great loss of EV-associated protein expression within 30 min after centrifuging and that low pH values may help reduce such degradation [36]. Nonetheless, more exhaustive information must be acquired for this concern. Another important note is that, upon storage, part of the isolated EVs may adhere to the storage container, resulting in a loss of material [8].

### Separation/concentration of EVs

In most literature-reported cases, EVs are usually isolated prior to their utilisation and study. They can be collected from EV-containing matrixes, such as biological fluids, in multiple ways, and many factors can deeply affect the recovery [37]. It is thus important to plan thoroughly this pre-analytical phase, keeping attention to storage and manipulation of the source material. Undoubtedly, a perfect isolation/purification of EVs is unrealistic and other non-vesicular structures may get co-isolated to various degrees [38]. Usually, the term “separation” is preferred to “isolation” and “purification”; other common expressions found in the literature and commercial kits are “enrichment” and “concentration”, which suggest an increase in EV counts relative to the total volume [8]. The decision in regard to what degree one should purify the EVs-containing sample depends on its experimental application. If there is no need to assess the biochemical composition or attribute a biomarker/function to a specific vesicle compared to others or to answer more complex clinical/analytical questions, then less pure EV-preparations can be considered, while focusing on higher recoveries [6, 8].

**Fig. 2:**
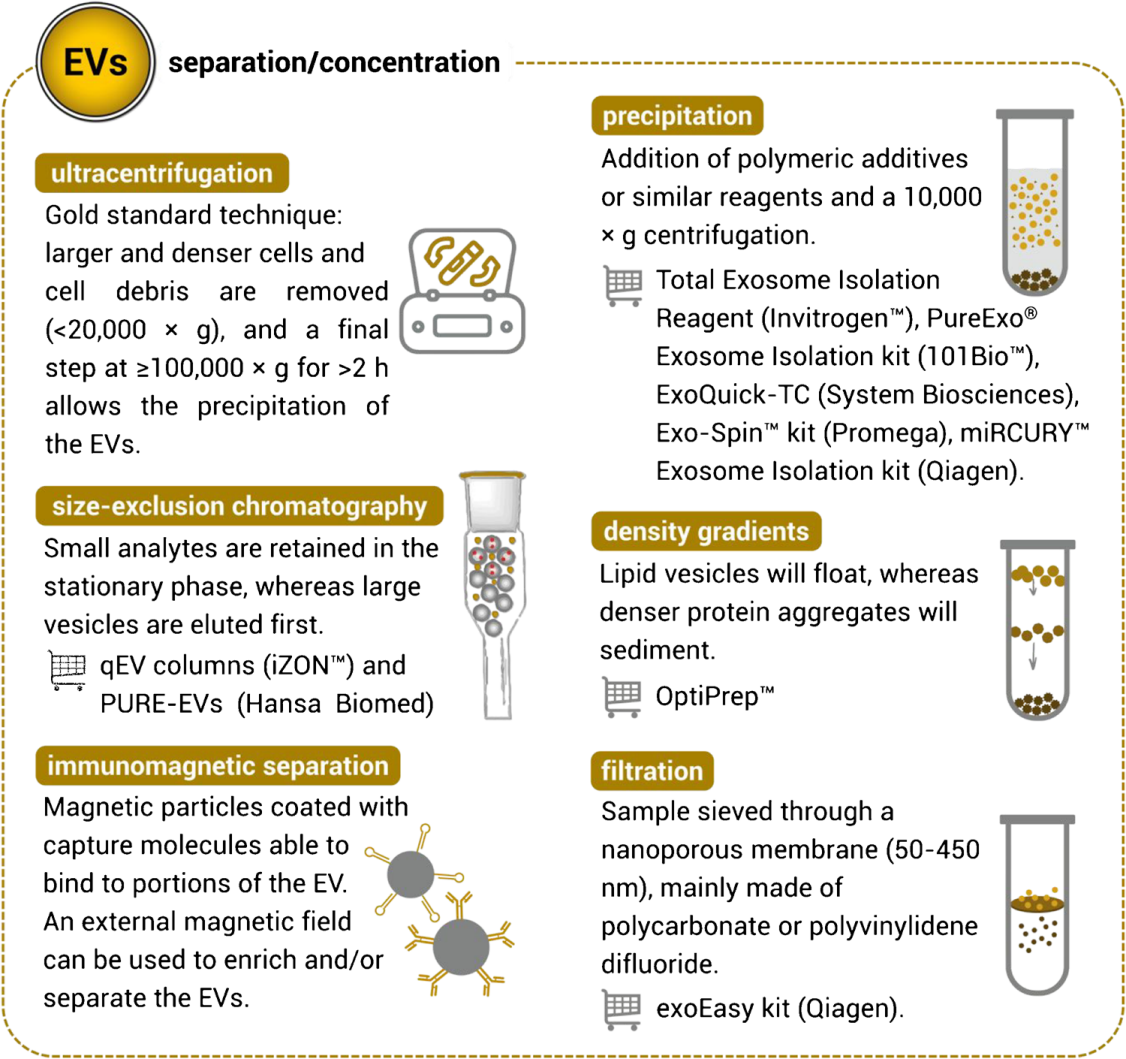
Most common approaches to achieve the separation or concentration of EVs from biological samples, with additional notes on widely used commercially available solutions to perform them

The methods used to separate EVs rely mainly on their properties, such as density, size, and surface markers. Many researchers use more than one separation/concentration step to obtain the final EV sample. According to the findings of C. Gardiner and colleagues [39], ultracentrifugation (UC) is the most commonly used first-step isolation methodology. Nonetheless, many other techniques can be found in the literature, such as density gradients [40], filtration [41], polymer-based precipitation [42, 43], immunoaffinity-based isolation [44], and size-exclusion chromatography [45], that, at the cost of some output impurities (e.g., co-precipitated proteins), may yield higher EV concentrations [46]. Figure 2 illustrates the various separation techniques often chosen by researchers, with a note on some of the kits and reagents available on the market. Indeed, each different approach presents its advantages and disadvantages. Since a standardisation among them has not yet been provided [47], case-by-case situations should be studied by a researcher that needs to adapt these procedures to the analytical question involved.

## Electrochemical detection of extracellular vesicles

When dealing with the detection of EVs, one can be interested in two different information: the total amount of EVs present, useful as an index of the presence of a tumour, or the specific identification of the type of cancer from which EVs originate. Interestingly, the membrane of EVs presents tetraspanins, proteases, transmembrane receptors, and adhesion molecules that may differentiate the vesicles depending on the parent cell. In the case of cancer-originating extracellular vesicles, the antigens present on their surface are considerably enriched, thus representing a great parameter to consider in the (*a*) detection and (*b*) identification of EVs. Indeed, one can consider different types of biomarkers that can be broadly categorised into two distinct applications: (*a*) universal cancer biomarkers, such as proteins abundantly present on the membrane of all EVs (although greatly enriched in cancer-derived EVs), useful when the objective is to quantify the whole set of particles present and detect their upregulation during on-going neoplasia; and (*b*) cancer-specific antigens, hence molecules that originate from certain tumours and may help identify it or distinguish it from others [48]. Besides membrane-tethered markers, EVs also contain an important cargo that includes a variety of biomolecules, such as proteins and nucleic acids [49]. As will be better discussed in the “Intravesicular biomarkers” section, the analysis of such intra-EV biomarkers is earning extensive attention in biosensing. A number of online-available databases, such as Vesiclepedia, EVpedia, and Exocarta, offer the possibility to check regularly updated information regarding the composition of EVs, thus representing valuable tools for researchers on the lookout for new detectable molecules that may help detect EVs or distinguish among them.

### EV detection using universal markers

Since it has been shown that their upregulation occurs in cancer cell lines upon their secretion into the extracellular space, EVs represent a valid diagnostic and prognostic biomarker [50]. The vast majority of research articles present in the literature carry out total EV detection by harnessing the presence of tetraspanins, such as CD9, CD63, CD81, and CD82, although CD81 and CD63 are by far the most exploited. In addition, lipid rafts, including cholesterol, ceramide, and phosphatidylserine, can also be used. Tetraspanins compose a superfamily of proteins characterised by four transmembrane domains. As abovementioned, they usually present widespread tissue distribution, but tissue-specific tetraspanins can also be found [51]. Their wide expression makes them the favourite candidate as the target in bioaffinity assays. Notwithstanding, tetraspanins and other widely expressed surface proteins are indeed often deficient in terms of specificity.

This section will discuss literature-reported examples that tackle total EV detection using ubiquitous markers (e.g., tetraspanins), categorising them by the type of biorecognition elements used for their capture, in label-free or label-based approaches, or even exploiting different capture mechanisms. Table 1 summarises all the crucial aspects behind the realisation of the biosensing platforms discussed in this section. Then, the section that follows will instead focalise on a more targeted recognition exploiting biomarkers that are specifically found in certain types of tumours.

**Table 1:** Electrochemical biosensors that perform total EV isolation and detection by using tetraspanins

Biomarker	Biorecognition element	Cell lines (cancer)	Isolation technique(s)	Signal amplification strategy	Electrochemical technique (electrode)	LOD (EVs/mL)	Dynamic range (EVs/mL)	Real samples	Ref
CD9	Antibody	MCF7 (breast)	UC	Nanomaterials	DPV (PCE)	7.0 × 10^4^	10^5^–10^10^	Spiked plasma	[52]
CD81	Antibody	MCF-7 (breast)	UC	None	EIS, DPV (GE)	77 (EIS), 379 (DPV)	10^2^–10^9^	No	[53]
CD63	Antibody	NS (NS)	Size-exclusion chromatography	None	EIS, EQCM-D (GE)	6.71 × 10^7^	NS	Spiked serum	[54]
CD9, CD63, CD81, CD24, CD44, CD54, CD326, CD340	Antibody	MCF-7, MDA-MB-231, SKBR3 (breast)	Differential UC, immunomagnetic separation	None	CA (m-GEC)	< 10^5^	NS	Serum of breast cancer patients	[55]
CD9, CD81, CD63, CD326, ALP	Antibody	hFOB	UC	None	(BDD)	10^8^	NS	Serum of breast cancer patients	[56]
CD81, CD63	Antibody	NS (NS)	Total EV isolation kit (Invitrogen)	None	SWV (GE)	10^5^	Up to 1.14 × 10^8^	Clinical urine samples	[57]
N-glycoprotein	Aptamer	SMMC-7721 (liver)	UC	None	DPV (GE)	1.5 × 10^6^	10^6^–10^8^	No	[60]
CD63	Aptamer	MCF-7 (breast)	UC	SiO_2_@Ag	DPV (GCE)	1.08 × 10^6^	4.2 × 10^5^ to 4.2 × 10^11^	Serum of breast cancer patients	[67]
CD63	Aptamer	MCF-7 (breast)	ExoQuick Exosome Isolation Kit (System Biosciences)	HCR, click chemistry	DPV (GCE)	96 × 10^3^	1.12 × 10^5^ to 1.12 × 10^11^	Serum of breast cancer patients	[61]
CD63	Aptamer	HeLa (cervix)	Centrifugation	Multipedal DNA walker strategy	SWV (GE)	6 × 10^3^	10^4^ to 2 × 10^6^	No	[62]
CD63	Aptamer	MCF-7 (breast)	Pre-enriched exosomes used	MOFs, dual signal output	SWV (ITO)	100	1.3 × 10^2^ to 2.6 × 10^5^	Plasma of breast cancer patients	[65]
CD63	Antibody and aptamer (sandwich)	HepG2 (liver)	exoEasy Maxi Kit (Qiagen)	CTSDR	SWV, EIS (GE)	1.72 × 10^4^	1 × 10^5^ to 5 × 10^7^	Human serum (10%)	[63]
CD63	Capture probe-free, aptamer for recognition	MCF-7 (breast)	Exo-Spin™ kit (Promega)	MOFs, HCR, DNAzymes	DPV (SPCE)	5 × 10^3^	1.7 × 10^4^ to 3.4 × 10^8^	Spiked 10% FBS	[66]

*ALP*, alkaline phosphatase; *BDD*, boron-doped diamond electrode; *CA*, chronoamperometry; *CTSDR*, cascade toehold-mediated strand displacement reaction; *DPV*, differential pulse voltammetry; *EIS*, electrochemical impedance spectroscopy; *EQCM-D*, electrochemical quartz crystal microbalance with dissipation monitoring; *FBS*, fetal bovine serum; *GCE*, glassy carbon electrode; *GE*, gold electrode; *HCR*, hybridisation chain reaction; *hFOB*, human fetal osteoblastic; *ITO*, indium-tin oxide; *m-GEC*, magneto-actuated graphite-epoxy composite electrode; *MOFs*, metal–organic frameworks; *NS*, not specified; *PCE*, paper-based carbon electrode; *SPCE*, screen-printed carbon electrode; *SWV*, square-wave voltammetry; *UC*, ultracentrifugation

#### Capture of EVs with antibodies

Although being the simplest and most common biorecognition elements, the scientific community is slowly trying to overcome antibodies due to some of their disadvantages, such as the need for in vivo laborious procedures for their obtainment. Nonetheless, antibodies are still greatly utilised in EV biosensing [52]. Kilic T. and co-workers [53], for instance, proposed a very simple label-free detection scheme involving the biorecognition reaction between an Anti-CD81 antibody and the CD81 present on the lipid membrane of the EVs, followed by EIS measurements. They obtained a LOD of 77 EVs/mL, with a dynamic range between 10^2^ and 10^9^ EVs/mL. Very recently, a similar label-free EIS-based immunosensing approach was studied by Guldin’s group [54].

**Fig. 3:**
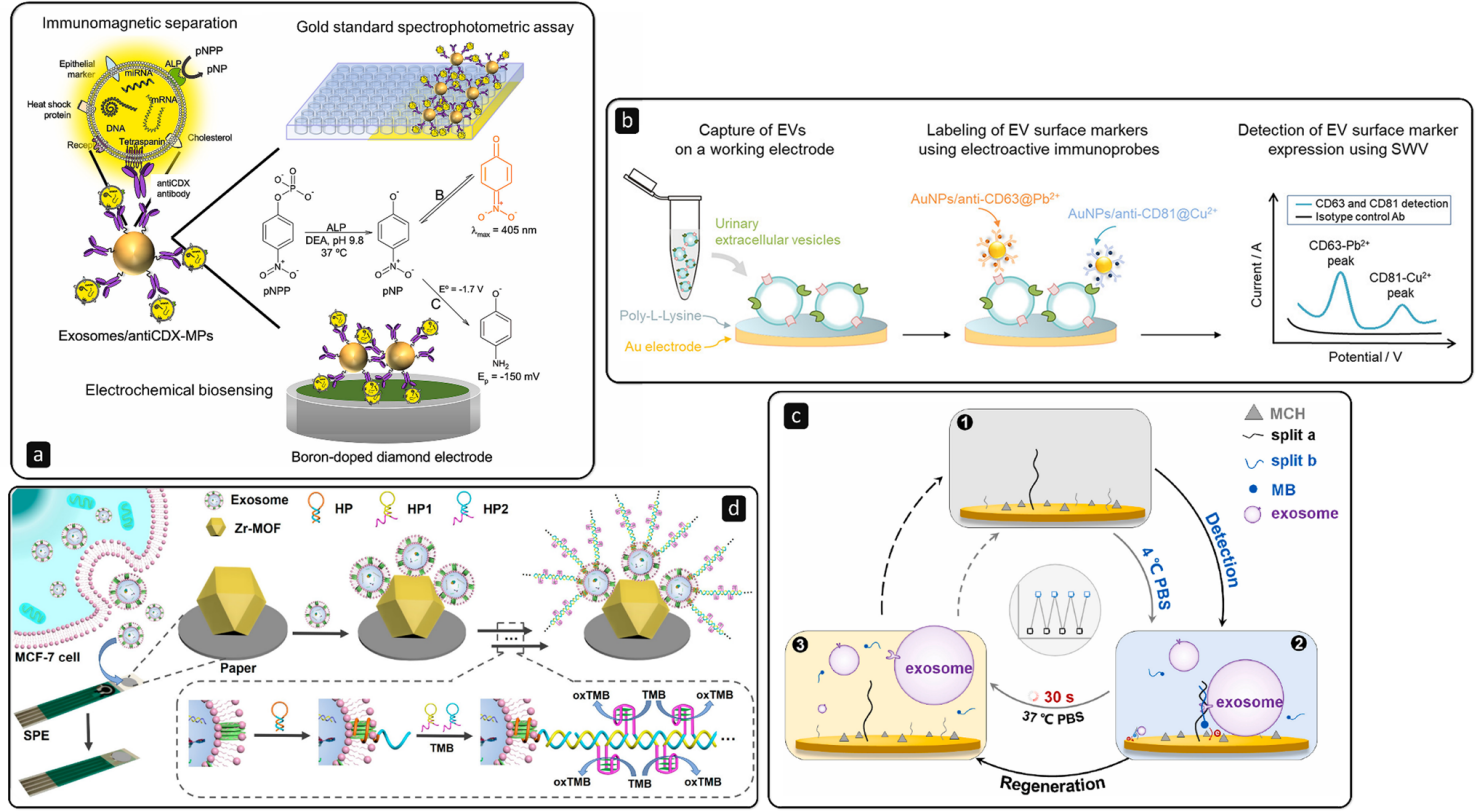
**a **Determination of immunomagnetically separated EVs through a colorimetric and electrochemical assay exploiting the enzymatic activity between the alkaline phosphatase (ALP) intrinsically present on EVs and *p*-nitrophenyl phosphate (pNPP) to produce electroactive *p*-nitrophenol (pNP); reprinted with permission from ref. [56]. **b **Capture of urinary EVs on a Au electrode and multiplexed square-wave voltammetry (SWV) detection by labelling with Au NPs functionalised with anti-CD63 and anti-CD81 antibodies doped with metal ions. Reprinted with permission from ref. [57], Copyright 2021 American Chemical Society. **c** The aptamer fragment split-a immobilised on a Au electrode recognised the N-glycoprotein on the surface of EVs, whereas a second fragment (split-b) was used for labelling with methylene blue (MB), but such configuration could be destroyed by rinsing the electrode in phosphate buffer saline (PBS) at 37 °C, thus allowing surface regeneration; reprinted with permission from ref. [60]. **d** EVs were adsorbed on Zr-MOFs-modified paper and then three hairpin probes (HP, HP1, HP2) recognised the CD63 marker on the membrane, triggering an HCR and amplifying the signal generated by using tetramethylbenzidine (TMB); reprinted with permission from ref. [66], Copyright 2021 American Chemical Society

Magnetic particles (MPs) functionalised with antibodies that recognise tetraspanins are widely available on the market from a variety of sources. Otherwise, researchers can easily functionalise MPs by harnessing a number of interactions (e.g., the streptavidin–biotin interaction, thus immobilising biotinylated antibodies on streptavidin-coated beads). Hence, MPs can be used to achieve a simple and fast pre-concentration of EVs even in complex biological media. Moura and co-workers [55] investigated the electrochemical detection of EVs through a capture based on MPs modified with different Anti-CDX antibodies. In particular, they focused on general tetraspanins (e.g., CD9, CD63, and CD81), but also on tetraspanins more specific in cancer-derived EVs (e.g., CD24, CD44, CD54, CD326, and CD340). The EVs were harvested from three breast cancer cell lines, namely MCF-7, MDA-MB-231, and SKBR3. They noticed that the separation based on Anti-CD81 antibodies was less affected by free receptors potentially present in the sample, hence resulting in an approach that could avoid UC to separate EVs from proteins. Moreover, studying serum samples from patients with breast cancer, they observed better performances when they combined the capture based on a specific cancer receptor (e.g., CD24 and CD340) and the following labelling with a second receptor focused on more general antigens (e.g., CD63). Very recently, the same group [56] proposed for the first time the electrochemical biosensing of EVs by exploiting the intrinsic presence of the enzyme alkaline phosphatase (ALP) on their surface (Fig. 3a). In particular, after capturing the EVs with Anti-CDX antibodies (i.e., CD81, CD63, and CD9), they monitored the activity of ALP by detecting the electroactive product generated through the enzymatic reaction with *p*-nitrophenyl phosphate (pNPP). Different types of antibodies can also be used in parallel. Revzin’s group [57] managed to simultaneously detect CD63 and CD81 biomarkers by functionalising Au nanoparticles (NPs) with anti-CD63 and anti-CD81 antibodies doped with metal ions (Pb^2+^ and Cu^2+^) to generate a redox signal (Fig. 3b). By doing this, they gained a sensitivity expressed with LODs around 10^5^ particles/mL.

#### Capture of EVs with nucleic acids

Nucleic acid–based detection uses the functional and structural properties of nucleic acids for the development of interesting approaches in the capture and detection of EVs. Aptamers, for instance, are single-stranded (ss) oligonucleotides whose three-dimensional conformation gives them the ability to bind to specific targets [58]. Indeed, researchers are constantly putting more effort into the development and improvement of aptasensors for the detection of EVs [59, 60] (Fig. 3c). When dealing with nucleic acids, one of the most common forms of signal amplification relies on the hybridisation chain reaction (HCR). It is an isothermal and enzyme-free technique that can be triggered when at two stable DNA monomers (DNA hairpins) in solution gets added a target DNA fragment. Thus, it can be easily initiated by using DNA aptamers when capturing EVs. An et al*.* [61] captured EVs on a glassy carbon electrode using an aptamer able to recognise CD63 on EVs after incubation at 37 °C for 1 h. To increase the number of immobilised aptamers, the electrode was modified with graphene oxide (GO) and Au NPs. Moreover, the signal was amplified through HCR and the electrochemical method was based on click chemistry, resulting in the ultrasensitive quantification of EVs by monitoring the reduction current of 2,3-diamoniphenazine. The practical applicability of the sensor was successfully tested in real serum samples derived from healthy individuals and breast cancer patients, proving to be resistant to interferences and promising in clinical diagnostics.

The molecular properties of nucleic acids (e.g., self-assembly) can indeed be of use to design biosensing nanostructures. Among these novel nanotechnologies, mainly used for signal amplification, DNA nanotetrahedron-assisted mechanisms have found applications in the field of EVs. Nowadays, DNA walker–based strategies are gaining continuously more momentum in research as a way to amplify the signal. Through strand displacement, nicking endonucleases, or DNAzymes, a DNA walker can be moved along oligonucleotide sequences, producing detectable single-stranded DNA fragments from the destruction of the DNA along its path. Miao P. and Tang Y. [62] managed to immobilise multiple walker strands on a single EV. The captures took place by using Fe_3_O_4_@Au magnetic nanoparticles modified with aptamers that recognised CD63 proteins. Such multipedal DNA walker strategy allowed a LOD of 6 particles/µL in buffer solution, using EVs obtained from HeLa cells through conventional centrifugation. HeLa cells were also compared with equal concentrations of MCF-10A cells, which express lower levels of CD63, showing that the system is capable of discriminating between the two.

#### Capture using both antibodies and nucleic acids

Sandwich-like designs that use both antibodies and aptamers for capture and signal generation can also be found. A sandwich-like structure of these two biorecognition elements was reported in the work of Cao Y. et al*.* [63]. A first enrichment of the EVs was carried out by using magnetic beads coated with anti-CD63 antibodies at 37 °C for 1 h. Then, the conjugate was recognised by CD63 aptamers in 0.5 h and a cascade toehold-mediated strand displacement reaction (CTSDR) was triggered. Such molecular machine provided a LOD of 1.72 × 10^4^ EVs/mL.

#### Metal–organic framework–based platforms

Metal–organic frameworks (MOFs) are interesting materials that offer great advantages in analytical chemistry due to the high specific surface area. They are especially useful to augment the number of the platform’s accommodable electroactive molecules, thanks to their high porosity. Zirconium-phosphate (ZIF-67) MOFs are gaining wide attention lately as a coordination chemistry-based interaction with EVs (electrostatic interaction between their phospholipid membrane and Zr^4+^) in substitution to the classic biological recognition [64]. Sun Y. and colleagues [65] focused on the detection of CD63 proteins on MCF-7 cells-derived EVs. Particularly, they developed a self-calibration sensor based on the assembly of black phosphorus nanosheets and ferrocene (Fc)-doped ZIF-67 MOFs on an indium-tin oxide (ITO) electrode coated with methylene blue–labelled CD63 aptamers. A dual-signal output was thus generated by Fc and methylene blue, allowing a LOD down to 100 EVs/mL. The system was also challenged by analysing clinical plasma samples from breast cancer patients.

Aptamers and antibodies are largely used as capture probes, as already shown. However, some assay schemes could benefit from the absence of capture probes, relying on different forms of immobilisation. For instance, hydrophilic phosphate heads present on the surface of EVs can be cleverly exploited for the direct enrichment of EVs without using other biorecognition elements. It is the case of X. Liu’s work [66], in which the authors used Zr-MOFs-functionalised paper for the coordinate interaction with the hydrophilic phosphate heads onto the surface of EVs (Fig. 3d). After such immobilisation, the signal was generated through the recognition mediated by CD63 aptamers and amplified via HCR, which generated multiple hemin/G-quadruples DNAzymes. Such architecture achieved a LOD of 5 × 10^3^ particles/mL.

### EV detection using cancer-specific markers

#### Surface-embedded biomarkers

Tetraspanins and the other markers mentioned before cannot always give specific information about the cancer involved. However, a wide variety of host cell–dependent proteins are also available on the EVs’ surface and can be of use to gather information on ongoing neoplasia [68]. More biomarkers can be specific for more types of tumours and vice versa [69]. The following section will discuss works that focus on some of the specific cancer markers found on the membrane that encloses the EVs. Table 2 gives a schematic outline of some of the aspects reported in the research papers mentioned, categorising them on the basis of the biomarker and the tumour studied.

**Table 2:** Electrochemical biosensors that target cancer-specific biomarkers present on the surface of EVs

Biomarker	Biorecognition element	Cell lines (cancer)	Isolation technique(s)	Signal amplification strategy	Electrochemical technique (electrode)	LOD (EVs/mL)	Dynamic range (EVs/mL)	Real samples	Ref
EGFR	Peptide	U87 (glioblastoma)	Differential centrifugation	MOFs	SWV (GE)	7.83 × 10^6^	9.5 × 10^6^ to 1.9 × 10^10^	Human serum	[72]
EGFR, CD9, CD63	Affibody	H1975 (NSCLC)	Size-exclusion chromatography	None	Electrokinetic detection	2.8 × 10^8^	0.8 × 10^9^ to 3.5 × 10^9^	No	[71]
MUC1 and CD63	Aptamer	MCF-7 (breast)	Exo-Spin™ kit (Promega)	HCR	DPV (ITO)	3.0 × 10^4^	1 × 10^5^ to 3.7 × 10^8^	Serum of breast cancer patients	[79]
CA-125 and CD9	Antibody	OVAR3 (ovary)	Total Exosome Isolation Reagent (Invitrogen)	None	DPV (PCE)	7.1 × 10^8^	NS	No	[80]
PTK7 and PSMA	Aptamer	CCRF-CEM (lymphoblastic leukemia)	Differential centrifugation	Cyclic enzymatic amplification	DPV (GE)	9.2 × 10^5^	4 × 10^6^ to 8 × 10^10^	No	[81]
EpCAM and CD63	Aptamer	MCF-7 (breast)	Centrifugation	3D-DNA walker	DPV (GE)	1.3 × 10^4^	5.0 × 10^4^ to 1.0 × 10^10^	FBS	[82]
EpCAM, CD9	Antibody	LNCaP (prostate)	Centrifugation	None	CV (GE)	3.0 × 10^5^	NS	Undiluted serum, urine	[77]
EpCAM and CD63	Aptamer	MCF-7 (breast)	Centrifugation + filtration	DNA walker, Ag@C nanocomposites	DPV (GE)	4.0 × 10^4^	10^5^ to 75 × 10^6^	30–60% FBS	[83]
EpCAM	Aptamer	MCF-7 (breast)	UC	mHCR	CA (GE)	2.85 × 10^5^	5 × 10^5^ to 1 × 10^8^	Human serum	[75]

*CA*, chronoamperometry; *CV*, cyclic voltammetry; *DPV*, differential pulse voltammetry; *EGFR*, epidermal growth factor receptor; *EIS*, electrochemical impedance spectroscopy; *EpCAM*, epithelial cell adhesion molecule; *FBS*, fetal bovine serum; *GE*, gold electrode; *HCR*, hybridisation chain reaction; *ITO*, indium-tin oxide; *LNCaP*, lymph node carcinoma of the prostate; *mHCR*, multidirectional HCR; *MOFs*, metal–organic frameworks; *MUC1*, mucin 1; *NSCLC*, non-small-cell lung cancer; *PCE*, paper-based carbon electrode; *PSMA*, prostate-specific membrane antigen; *PTK7*, tyrosine kinase–like 7; *RCA*, rolling circle replication; *SWV*, square-wave voltammetry; *UC*, ultracentrifugation

##### **Epidermal growth factor receptor (EGFR)**

Human EGFR is highly expressed in glioblastoma-derived EVs, thus representing an optimal non-invasive candidate for the early detection of such intracranial tumour. Instead of antibodies and aptamers, anti-EGFR affibodies, namely affinity reagents able to bind to a variety of targets [70], have been successfully used to functionalise microcapillaries and capture EVs harvested from non-small-cell lung cancer (NSCLC) cell lines H1975 in a label-free fashion [71]. Besides avoiding labelling probes and agents, bypassing the need for capture probes is also desirable. As introduced in the previous section, Zr-MOFs show a high affinity to phosphate groups, forming Zr-O-P interactions. Thanks to these bonds, EVs can be captured or recognised without the need for specific probes, but by exploiting the intrinsic phosphate groups present on their surface. Sun Z. et al*.* [72], for instance, immobilised glioblastoma-derived EVs, obtained by differential centrifugation from U87 cell lines, using a peptide ligand able to selectively bind to the EGFR. Then, the electrochemical signal is generated by the absorption of methylene blue–loaded Zr-MOFs. The researchers obtained a LOD of 7.83 × 10^3^ EVs/µL with this method. Indeed, peptides have shown to be interesting synthetic recognition elements with good binding affinities and low costs [73, 74]. Moreover, their synthesis is easily implemented with unpaired production rates. Their tuneable functional groups can be conveniently used to target multiple biomolecules, hence representing a versatile tool in biosensing. However, to the best of our knowledge, only very few examples of EV detection using peptides can be found in the recent literature.

##### Epithelial cell adhesion molecule (EpCAM)

EpCAM, sometimes indicated as CD326, is a transmembrane glycoprotein frequently heterogeneously overexpressed in carcinomas, but not in cancers of non-epithelial origin. Chen’s group [75] recently introduced a multidirectional HCR (mHCR) to enhance the detection of EVs from MCF-7 cells using aptamers with an affinity for the EpCAM biomarker for their capture. Notably, to trigger mHCR, they used a cholesterol-modified H shape–like DNA structure able to plunge its cholesterol moiety into the lipid bilayer of EVs, thus getting anchored. The signal, measured via chronoamperometry, was generated by the redox reaction of 3,3′,5,5′-tetramethylbenzidine (TMB) and hydrogen peroxide (H_2_O_2_) catalysed by horseradish peroxidase (HRP). The authors reported a LOD of 285 EVs/µL and managed to discriminate between tumorous (MCF-7 cells) and non-tumorous (MCF-10A cells) EVs in human serum. This example highlights the potential of lipid anchors, such as cholesterol, as new biorecognition elements that can be inserted, via hydrophobic interactions, into the lipid bilayer of the EV’s membrane. This is especially interesting since such recognition is not influenced by the number of markers present on the surface of EVs [76].

**Fig. 4:**
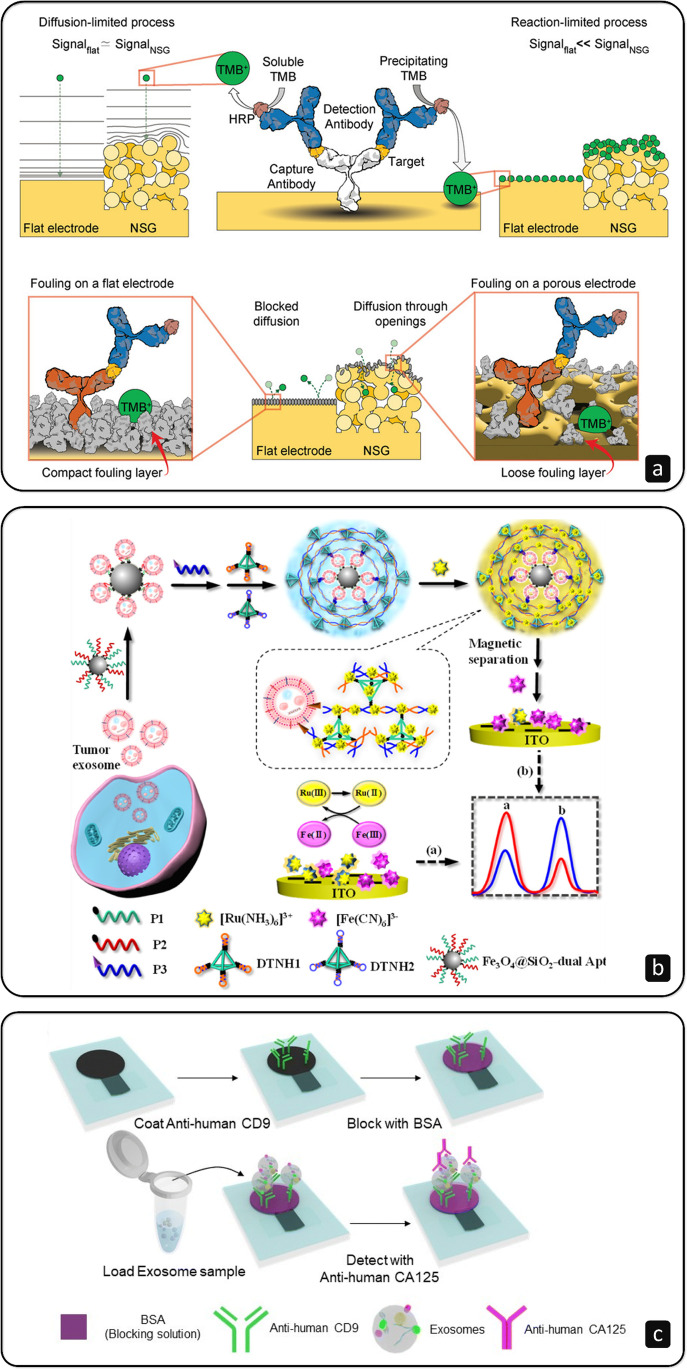
**a** Signal output and fouling effect between a flat electrode surface and a nanoporous gold (NSG) electrode using tetramethylbenzidine (TMB) as electroactive compound in the analysis of undiluted biological samples; reprinted with permission from ref. [77]. **b** Dual-aptamer simultaneous recognition of MUC1 (P1) and CD63 (P2) markers on magnetically separated EVs, amplifying the signal through hyperbranched HCR mediated by a cholesterol-modified DNA probe (P3) and DNA tetrahedral nanostructures (DTNH1 and DTNH2); reprinted with permission from ref. [79], Copyright 2021 American Chemical Society. **c** Sandwich electrochemical immunoassay for the analysis of ovarian cancer cell–derived EVs using CD9 and CA-125 antibodies on paper-based carbon electrodes (PCEs); reprinted with permission from ref. [80]

When dealing with complex biological fluids, the analysis often faces hurdles linked to nonspecific adsorption of unwanted material (e.g., proteins) on the electrode surface, thus limiting the electroactive area available and, therefore, the sensitivity of the assay. This is especially true in the case of non-isolated EVs, which represent a minor subpopulation of the whole set of circulating particles found in such biological media. In this regard, nanoporous structures could be beneficial by limiting the diffusion and adsorption of fouling material [52], as investigated in a recent work that captures prostate cancer EVs from undiluted plasma and urine samples using antibodies (Fig. 4a) [77].

##### Mucins

**Fig. 5:**
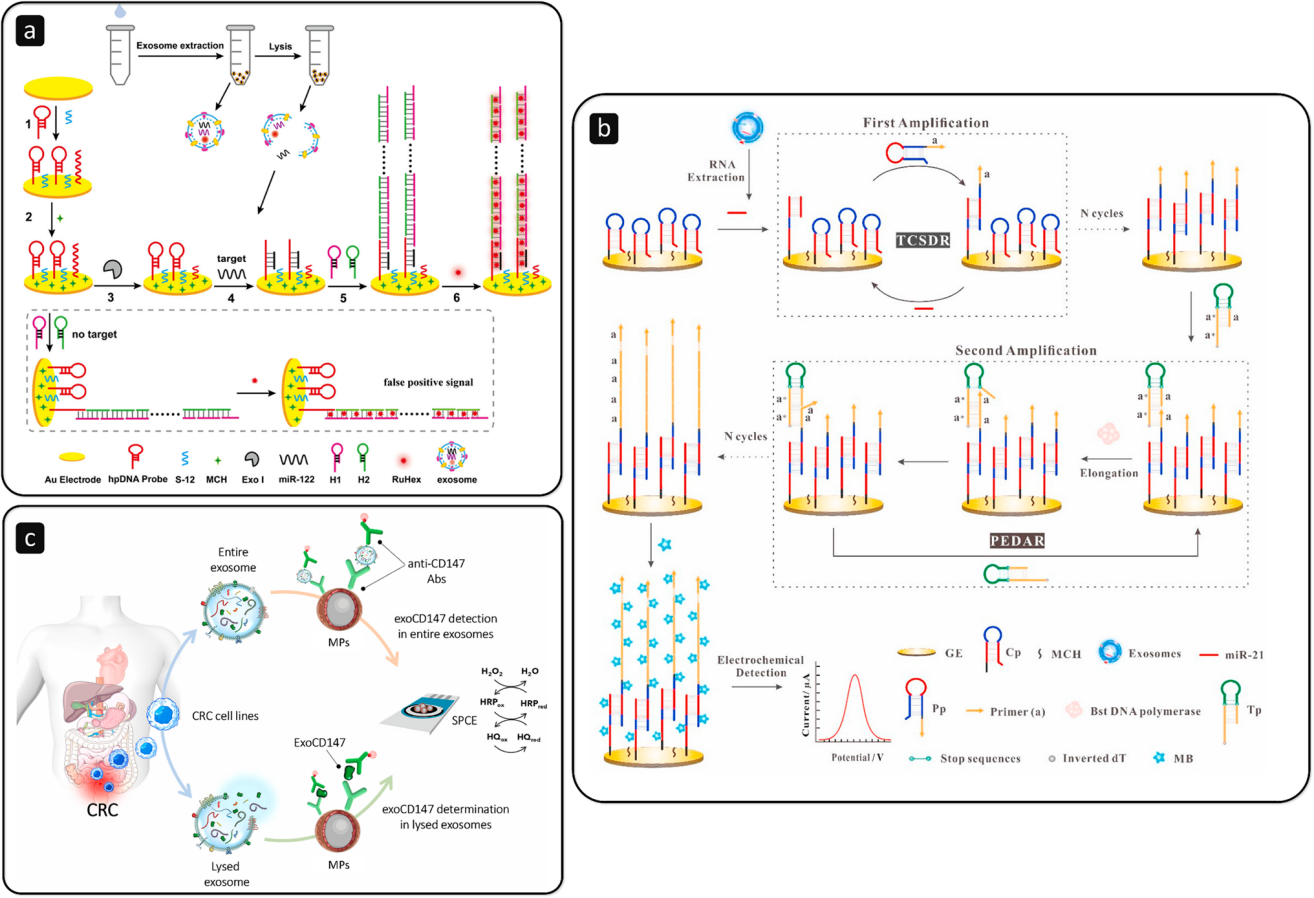
**a** Extraction and lysis of EVs to capture intravesicular microRNA-122 using a hairpin DNA (hpDNA) probe immobilised on a Au electrode and triggering HCR using DNA helpers (H1 and H2) to amplify the signal generated by Ru(NH_3_)_6_.^3+^ (RuHex); exonuclease I (Exo I) was used to eliminate false positive signals; reprinted with permission from ref. [91], Copyright 2020 American Chemical Society. **b** Dual-amplification reactions by target-mediated cyclic strand displacement reaction (TCSDR) and primer exchange DNA amplification reaction (PEDAR) for the detection of miRNA-21 in the presence of primer probes (Pp); template probes (Tp) hybridise with the TCSDR-generated primers and PEDAR continues with the assistance of Bst DNA polymerase; finally, the signal is generated using methylene blue (MB); reprinted with permission from ref. [94]. **c** Detection of the CD147 biomarker in intact and lysed EVs derived from colorectal cancer (CRC) cell lines with a sandwich of antibodies onto the surface of MPs immobilised on screen-printed carbon electrodes (SPCE), and the subsequent amperometric transduction using hydroquinone (HQ) and H_2_O_2_ in the presence of horseradish peroxidase (HRP); reprinted with permission from ref. [97]

The mucin family is composed of glycosylated macromolecules largely expressed in mammalian epithelial cells [78]. It has been reported that the abnormal expression of Mucin 1 (MUC1), a high molecular weight membrane glycoprotein, can represent a potential cancer marker. Yang L. and colleagues [79] recently proposed a dual-aptamer isolation and recognition of EVs from MCF-7 cell lines exploiting the overexpression of both MUC1 and CD63 markers on their surface (Fig. 5b). After the capture, a cholesterol-modified DNA probe could be anchored onto the EVs and trigger an HCR for signal generation and amplification. Mucin 16, also known as the cancer antigen 125 (CA-125), is the most prominent EV biomarker in the case of ovarian cancer [78]. Very recently, Kasetsirikul S. et al. [80] worked on the development of a paper-based device able to capture and detect the whole bulk of EVs using antibodies that bind to generic markers (e.g., CD9) (Fig. 4c). Then, they used antibodies specific to the CA-125 marker to identify ovarian cancer–positive samples (LOD of 7.1 × 10^8^ EVs/mL).

##### Tyrosine kinase–like 7 (PTK7) and prostate-specific membrane antigen (PSMA)

PSMA and PTK7 are two abundantly expressed proteins on the membrane of EVs harvested from LNCaP and HeLa cell lines, respectively. Recently, Yu Y. and co-workers [81] focused on both biomarkers and used them as inputs for an and logic gate assisted by dual-aptamer recognition and cyclic enzymatic signal amplification. With this approach, they managed to improve the specific recognition and lower interferences. In each microliter of sample, they could detect down to 920 EVs obtained from CCRF-CEM cells (human leukemic lymphoblasts) by differential centrifugation.

#### Intravesicular biomarkers

Indeed, most electrochemical biosensors are used for the detection of surface-embedded protein markers on EVs. However, EVs also inherit important cargo molecules (e.g., intravesicular nucleic acids) that can be of clinical use, keeping in mind that their biochemical composition highly depends on their physiological role, as well as their origin and fate. Many papers that include biorecognition molecules that are used to interrogate EV-derived microRNAs (herein abbreviated as miRNAs or miR) and other nucleic acids have been published. miRNAs from EVs have a role in cancer progression and metastatic events via intercellular communication: such encapsulated RNAs are transferred from cell to cell as a signalling method [84]. High levels of such nucleic acids (miRNA-21 is the most widely studied) may indicate poor survivability rates. Therefore, the analysis of intra-EV miRNAs may represent truly remarkable potential in cancer diagnostics. Interestingly, it is only in 2016 when the first product aimed at isolating and analysing exosomal RNA was released on the market in the USA [85].

The use of detergents and organic extractions are the most common solutions to induce the lysis of the vesicles, hence allowing access to the biomarkers present in the intravesicle space. Many commercial kits are available on the market to meet the need for simple and rapid ways to examine the molecular cargo (nucleic acids and proteins) of pre-enriched EVs. The most widely known is probably the TRIzol™ reagent (Life Technologies), introduced in 1987 by P. Chomczynski and N. Sacchi to isolate RNA from cells and tissues with a single extraction [86]. Similarly, the “Total Exosome RNA & Protein Isolation Kit” by Life Technologies follows three phases based on (*i*) an organic extraction with acid phenol:chloroform, (*ii*) a purification step by immobilising RNA on glass-fiber filters, and finally (*iii*) the elution using a low ionic-strength solution. The whole procedure takes about 30–60 min. Another widely used kit is the “exoRNeasy Midi/Maxi Kit” (Qiagen), which uses a membrane-based affinity binding step to recover the entire spectrum of EVs present in the sample, regardless of particular epitopes. The elution step is, again, based on a phenol and guanidine thiocyanate lysis to collect RNA (and denature RNases), following an organic extraction in chloroform. Table 3 provides a series of information regarding the research articles discussed in this section, including the lysis approach chosen.

**Table 3:** Electrochemical platforms that focus on the detection of the intravesicular cargo

miR/cell lines (cancer)	Capture	EV isolation technique	EV lysis approach	Signal amplification strategy	Electrochemical technique (electrode)	LOD	Dynamic range	Real samples	Ref
miR 21-5p/NS	Complementary probe	NS	NS	None	EIS (SPCE)	4.31 aM	0.01–10^4^ fM	Human serum	[87]
miR 200b/PNT1A, 22Rv1, LNCaP, PC3 (prostate)	Complementary probe	UC	Triton X-100 lysis buffer	None	EIS, SWV (GE)	122 aM	10^2^–10^8^ aM	Urine of prostate cancer patients	[88]
miR-21/SKBR-3 (breast), HKESC-1 (oesophagus), and SW-48 (colon)	Complementary probe	Total exosome isolation reagent (Life Technologies)	Total exosome RNA & protein isolation kit (Life Technologies)	None	DPV (GE)	1.0 pM	1–100 pM	Serum of colorectal adenocarcinoma patients	[98]
miR-21/NS (breast)	LNA	exoEasy Maxi Kit (Qiagen)	exoRNeasy Midi/Maxi Kit (Qiagen)	TSDR, RCA, G-quadruplex	DPV (GE)	2.75 fM	10–10^7^ fM	Plasma of breast cancer patients	[90]
miR-21/MCF-7 (breast)	LNA	UC	TRIzol™ reagent	TSDR	DPV (GE)	2.3 fM	10–70 fM	No	[99]
miR-21/MCF-7 (breast)	LNA	UC	TRIzol™ reagent	DNA walker, TSDR	DPV (GE)	67 aM	0.1–100 fM	Serum, cancer patients’ cells lines	[93]
miR-122/HepG2 (liver), MCF-7 (breast)	hpDNA	UC	TRIzol™ reagent	HCR	DPV (GE)	53 aM	0.1–10^8^ fM	No	[91]
miR-21/MCF-7 (breast)	hpDNA	UC	NS	HCR	CA, EIS (GE)	168 aM	NS	No	[92]
miR-21/MCF-7 (breast)	hpDNA	UC	Exosome RNA Isolation Kit (Rengen Biosciences Co.)	TCSDR, PEDAR	SWV (GE)	3.04 aM	10–10^7^ aM	Serum of breast cancer patients	[94]
miR-21/MCF-7 (breast), HeLa (cervix)	PNA	ExoQuick-TC (System Biosciences)	exoRNeasy serum/plasma Midi Kit (Qiagen)	None	SWV, CC (GE)	49 aM	10^2^–10^9^ aM	Spiked human serum, patients’ peripheral blood plasma	[96]
miR-21/MCF-7 (breast), HeLa (cervix), A549 (lung)	PNA	ExoQuick-TC (System Biosciences)	exoRNeasy serum/plasma Midi Kit (Qiagen)	TDN	CC (GE)	34 aM	10^2^–10^9^ aM	Plasma	[95]
CD147/SW480 (colon)	Antibody	UC	RIPA buffer (Sigma-Aldrich)	None	CA (SPCE)	29 pg/mL	0.096–5.0 ng/mL	None	[97]

*CA*, chronoamperometry; *CC*, chronocoulometry; *DPV*, differential pulse voltammetry; *EIS*, electrochemical impedance spectroscopy; *GE*, gold electrode; *HCR*, hybridisation chain reaction; *HKESC-1*, Hong Kong esophageal squamous carcinoma-1; *hpDNA*, hairpin DNA probe; *LNA*, locked nucleic acid; *miR*, microRNA; *NS*, not specified; *PEDAR*, primer exchange DNA amplification reaction; *PNA*, peptide nucleic acid; *RCA*, rolling circle replication; *RIPA*, radioimmunoprecipitation assay; *SPCE*, screen-printed carbon electrode; *SWV*, square-wave voltammetry; *TCSDR*, target-triggered cyclic strand displacement reaction; *TDN*, tetrahedral DNA nanolabel; *TSDR*, toehold-mediated strand displacement reaction; *UC*, ultracentrifugation

Instead of antibodies and aptamers, complementary sequences of DNA are commonly used as bioreceptors in miRNA analysis [87, 88]. Magnetic particles have been applied for years in the field of nucleic acids [89]; thus, it is not unusual to find their implementation in the determination of EV-derived miRNAs [90]. Many approaches are used to enhance the sensitivity of the biosensing scheme, including signal amplification strategies like HCR [91, 92] (Fig. 5a) and molecular tools based on strand displacement reactions [93, 94] (Fig. 5b). Besides DNA sequences, other synthetic receptors are widely used. Peptide nucleic acids (PNA), for instance, are an interesting alternative [95]. The combination of a short electroneutral PNA probe, able to recognise the complementary domain in miRNA-21, with a spherical nucleic acid (SNA) nanoprobe, able to load great quantities of electroactive tags onto the electrode, was reported [96]. Such PNA-miRNA-SNA sandwich allowed the enzyme-free detection of the analyte down to concentrations of 49 aM.

As mentioned in the “Introduction”, the intravesicular cargo comprises multiple macromolecules in addition to nucleic acids. An interesting one, for instance, is the cluster of differentiation 147 (CD147), poorly studied up until now. It plays a crucial role, for instance, in the progression of colorectal cancer. Inspired by its relevance and the lack of articles that explore its detection, the research group of S. Campuzano [97] contributed by designing an electrochemical strategy based on a sandwich of antibodies able to capture the CD147 biomarker in EVs from colorectal cancer cells. In particular, using magnetic beads, they managed to detect such analyte both in intact and in lysed EVs (Fig. 5c), with results in agreement with those obtained by ELISA and Western blot techniques.

### Integrated lab-on-a-chip microfluidic platforms

Microfluidics allows the manipulation of small volumes of samples by means of precise fluidic control. The increasing number of integrated analytical platforms that comprise multiple analytical phases, from sample processing to detection, resulted in augmented research around microfluidic approaches to obtain EV separation on a portable scale [100]. After the first successful attempt in 2010 [101], in which it was shown that a microfluidic EV isolation without a previous UC step was possible working in serum, many researchers tried to implement this kind of separation strategy inside their biosensing platforms. Electrochemical sensors are easily integrated with microfluidics, thus obtaining rapid and cost-effective platforms apt for point-of-care testing (POCT) that include all the steps required in EV detection [102].

**Fig. 6:**
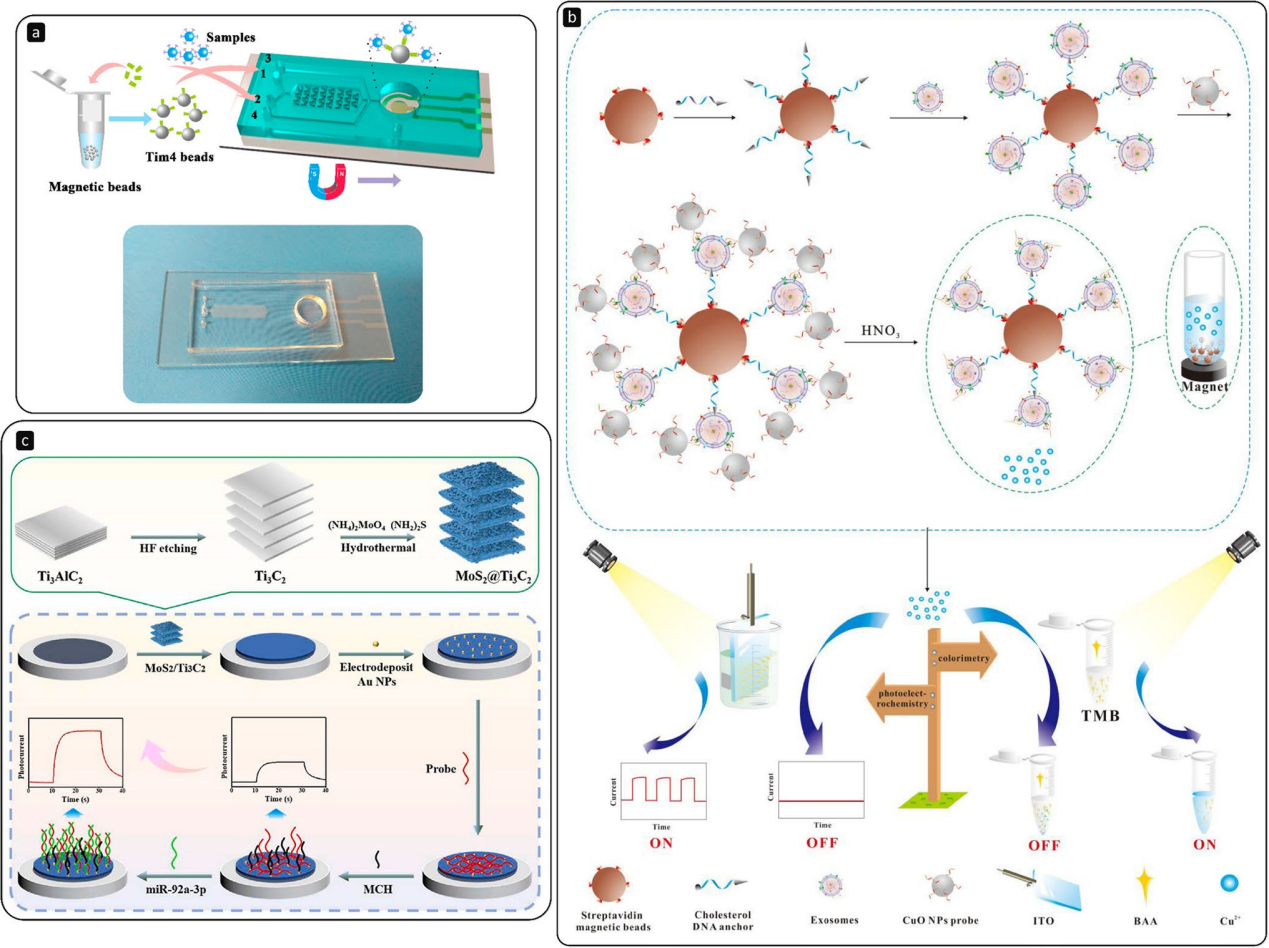
**a** Microfluidic platform that integrates magnetic separation of EVs, using Tim4-modified MPs, and electrochemical analysis on an ITO electrode; reprinted with permission from ref. [103], Copyright 2018 American Chemical Society. **b** Streptavidin-coated MPs modified with cholesterol DNA anchors to capture EVs through hydrophobic interactions, while a sandwich is formed using MUC1 aptamers modified with CuO nanoparticles (NPs) able to generate a change in absorbance and photocurrent response of 10-benzyl-2-amino-acridone (BAA) used as signalling probe; reprinted with permission from ref. [116]. **c** Au NPs deposited on a MoS_2_@Ti_3_C_2_ nanohybrid–based PEC platform for the analysis of EV-derived miRNA-92a-3p, using 6-mercapto-1-hexanol (MCH) to block nonspecific adsorption sites; reprinted with permission from ref. [120]

Among the diverse technologies exploited to isolate EVs, immunoaffinity-based separations can be implemented inside these chips, for instance by modifying the various microchannels with antibodies or inserting MPs functionalised with specific ligands into the flow. Xu H. et al. [103] presented a two-stage microfluidic platform that comprises both an on-chip magnetic isolation and a subsequent electrochemical detection of EVs (Fig. 6a). Interestingly, the capture is accomplished by using MPs modified with Tim4 receptors that bind to the phosphatidylserine expressed on the EVs’ membrane. The various liquids were injected using a programmable syringe pump at constant flow rates. A permanent magnet placed underneath the platform was used to retain the beads in position as the EV-containing solutions flowed. Then, the magnet was moved to the electrode area for the detection (LOD of 4.39 × 10^3^ EVs/mL). The device was able to discriminate between healthy and hepatic carcinoma patients, with a protocol that required 30 µL of clinical blood samples and took about 3.5 h for the response. In many immunoaffinity-based methodologies, the antibodies used to capture EVs are immobilised harnessing the high-affinity binding between biotin and avidin. However, if one needs to release the EVs captured for further downstream analyses, such strong binding is not ideal, since the operations required to break it operate at extreme pH and high temperature [104]. As an alternative to biotin, Nagrath’s group [105] worked with desthiobiotin, an analogue of biotin that presents a lower binding affinity to avidin, making it possible to release the EVs captured onto magnetic beads by EpCAM desthiobiotin-conjugated antibodies inside their microfluidic device.

Another isolation strategy is accomplished by size-based separations, for instance by adding membranes or nanowires with pore sizes apt for the filtration of particles with desired dimensions. A third option is characterised by the use of external forces between the flow medium and EVs therein contained, thus dynamically concentrating the particles in the light of their size, density, or other properties. Regarding this latter methodology, acoustic waves, electric fields, and centrifugal forces are the most common external inputs to achieve separation [106]. On the other hand, dynamic isolation is also possible relying solely on the hydrodynamic properties generated by the fluidic system. Deterministic lateral displacement (DLD) is indeed the most widespread design among these “passive” dynamic approaches. Briefly, in DLD, different flow laminae separate particles smaller and larger than a defined critical diameter [107]. Indeed, in microfluidic devices, sample mixing is pivotal and can be achieved by playing with the diffusion effect and creating different flows [108]. To enhance flow mixing, increase interactions between analytes and recognition elements, and augment the capture possibility of EVs, herringbone structures are widely used due to the chaotic effect they generate [109]. A geometrically activated surface interaction chip was implemented inside a detachable microfluidic device featuring an asymmetric herringbone structure to enhance the collision between cancerous EVs and biorecognition elements immobilised on a gold electrode [110]. In particular, EpCAM aptamers were used both for capture and labelling, achieving a LOD of 17 EVs/µL, with a dynamic range between 10^2^ and 10^9^ EVs/µL. Moreover, such detachable 3D-printed housing can be separated from the aptasensor, and EVs can be harvested for further downstream analyses. Exploiting a similar herringbone microfluidic mixer, Zhang Y. et al*.* [109] proposed a chip able to purify pancreatic cancer EVs directly from plasma, focusing on the glypican-1 biomarker. Then, the obtained EVs were also used to identify a miRNA signature in this kind of cancer, finding that miRNA-125b-5p and miRNA-214-3p could be of diagnostic value.

In the last decade, many complete critical reviews discuss thoroughly the application of microfluidics for isolation and analysis of EVs from biological fluids [9, 106, 111, 112]. It is interesting to point out that with such fluidic handling, EVs can maintain their original morphology; this is not always true for UC: the high mechanical force put on the particles tends to aggregate them [113]. Nonetheless, it has to be mentioned that microfluidics has yet to be improved, as for now, it cannot represent a stand-alone approach. As a matter of fact, architectures that run a microfluidic separation of EVs usually also include other techniques for better isolation [6].

## Photoelectrochemical detection of extracellular vesicles

With photoelectrochemical (PEC) methods, the advantages listed for electrochemical approaches add up to many others. Indeed, using photoexcitation, PEC schemes couple the merits of electrochemical and optical strategies, resulting in weak background noises, high sensitivities, low costs, and the possibility to be implemented in miniaturised devices with ease [114]. Unfortunately, to the best of our knowledge, very few research articles involving the use of PEC for the detection of EVs are present in the recent literature [115]. In one of the latest published [116], a biotinylated cholesterol DNA anchor was attached to streptavidin-coated MPs to obtain a structure that could act as a capture probe for EVs via hydrophobic interactions between cholesterol and the lipid bilayer of the EVs (Fig. 6b). The modified MPs were thus incubated with EVs derived from MCF-7 and MCF-10A cell lines for 60 min at + 4 °C, continuously shaking gently. Then, an incubation with CuO nanoparticles modified with a thiolated MUC1 aptamer (Cu–S bond) followed, forming a sandwich (MBs-EV-CuO NPs). The subsequent addition of HNO_3_ caused the hydrolysis of CuO NPs to Cu^2+^. A 10-benzyl-2-amino-acridone (BAA)-modified ITO electrode was used for photoelectrochemical measurements. Among others, acridone derivatives have photoelectrochemical properties under UV–vis illumination. They found out that Cu^2+^ inhibited the photocurrent of BAA, producing a signal decrease proportional to the concentration of EVs in the sample (LOD of 1.38 × 10^3^ particles/µL). This phenomenon was also confirmed by UV–vis spectroscopy and a colorimetric approach. Real clinical samples, particularly breast cancer patients’ serum, were also analysed to test the practical capability of the sensor. A similar capture approach based on a cholesterol-labelled DNA probe, but coupled to HCR, was also recently used to design a PEC biosensor able to detect EVs immobilised on magnetic beads modified with CD63 aptamers (LOD of 7.875 × 10^4^ EVs/mL) [117].

On the other hand, PEC analysis of nucleic acids is more robust and common [118]. Thus, compared to the detection of intact EVs, a wider number of research papers that consider the interrogation of intravesicular miRNAs can be found in the literature [119]. With PEC, the key is working with stable photoactive nanomaterials. Indeed, these elements play crucial roles, increasing the sensitivity of the assay and working as photoactive sensitisers. In addition, hybrid structures can be formed to achieve better performances. Very recently, Sun Z. and colleagues [120] fabricated a PEC sensor depositing Au NPs on a MoS_2_@Ti_3_C_2_ nanohybrid (Fig. 6c). By doing this, they immobilised DNA probes to detect miRNA-92a-3p obtained from EVs with the miRNeasy Micro Kit (Qiagen) derived from colorectal cancer patients (LOD of 0.27 fM). Wang Y. et al*.* [121] focused instead on a heterostructure based on WO_3_ nanoflakes coated with ZIF-67 MOFs for the PEC detection of miRNA-21 (LOD of 0.5 fM) using the release of hemin to trigger a photocurrent quenching in the presence of the analyte.

## Conclusions and future perspectives

The analysis of EVs’ content and surface allows precise conclusions regarding the cells from which they originated, as well as potential pathological conditions. By the examples discussed in the present review, electrochemical biosensors have recently proved their validity toward ultra-low determinations of EVs with the prospect of early-detections in cancer management. Examples of the (photo)electrochemical detection of intravesicular and extravesicular markers have been herein discussed, involving a wide variety of separation procedures, manipulation approaches, signal amplification strategies, and biorecognition elements. Researchers that work in this unstandardised field require constant updates to the recent procedures and their evolutions since EVs are analytes whose utilisation is often not straightforward.

Most scientific papers found in the literature use bioreceptors, e.g., antibodies and aptamers, that bind to the CD63 tetraspanin on EVs’ surface. Regarding aptamers, in particular, it is curious that most researchers use Anti-CD63 sequences and very few Anti-CD81 or Anti-CD9 aptamers, despite the fact that the latter two markers are quite common. When focusing on the molecular cargo of EVs, almost every work found in recent years uses miRNA-21 as model target, probably due to the vast knowledge accumulated over the years on this particular cancer biomarker, even though many other miRNA sequences can be found in EVs. However, the research around EV determination is still in its very first stages. The number of potential clinical biomarkers continuously increases, but their applications in the field are still quite limited. Indeed, many obstacles hamper clinical uses of the wide variety of biomarkers increasingly discovered nowadays, not least the low concentration and stability of these molecules inside biological fluids. More studies in this field will grant better knowledge about EV composition and its variation (e.g., due to the progression of the tumour), thus allowing more precise solutions for isolation and detection. Indeed, one of the main hurdles that has to be addressed in the near future regards the stability of EVs, in order to provide easier and more tailorable analyses. In addition to this, due to the heterogeneity in EV markers, multiplex analyses that focus simultaneously on more EV markers (both outside and inside) are highly desirable to provide accurate profiling of EVs in clinical samples. Furthermore, analytical workflows may be simplified, hence reducing the time required for the overall analysis. It is also crucial to highlight that, nowadays, the conventional methods described in the previous sections are difficult to implement at a clinical level due to their complexity and cost.

In our opinion, next-generation biosensing devices will be based on portable analytical tools and the scientific community is constantly moving toward this objective. After all, the COVID-19 pandemic has shown the true potential of POCT devices in diagnostics. Although not easy, we can envisage more and better-integrated platforms capable of seamlessly performing both separation and detection of EVs in near-patient applications. Magnetic beads could be an interesting tool able to easily perform EV isolation and then be magnetically immobilised onto the electrode for the detection, all in a straightforward workflow.
